# Comparing and Combining Artificial Intelligence and Spectral/Statistical Approaches for Elevating Prostate Cancer Assessment in a Biparametric MRI: A Pilot Study

**DOI:** 10.3390/diagnostics15050625

**Published:** 2025-03-05

**Authors:** Rulon Mayer, Yuan Yuan, Jayaram Udupa, Baris Turkbey, Peter Choyke, Dong Han, Haibo Lin, Charles B. Simone

**Affiliations:** 1 Oncoscore, Garrett Park, MD 20896, USA; 2 School of Computer Science, Faculty of Engineering, The University of Sydney, Sydney, NSW 2050, Australia; yyua9990@uni.sydney.edu.au; 3 Perelman School of Medicine, University of Pennsylvania, Philadelphia, PA 19104, USA; jay@pennmedicine.upenn.edu; 4 National Institutes of Health, Bethesda, MD 20892, USA; ismail.turkbey@nih.gov (B.T.); pchoyke@mail.nih.gov (P.C.); 5 New York Proton Center, New York, NY 10035, USA; dhan@nyproton.com (D.H.); hlin@nyproton.com (H.L.); csimone@nyproton.com (C.B.S.II)

**Keywords:** prostate cancer, biparametric MRI, spectral/statistical approaches, deep learning, artificial intelligence

## Abstract

**Background:** Prostate cancer management optimally requires non-invasive, objective, quantitative, accurate evaluation of prostate tumors. The current research applies visual inspection and quantitative approaches, such as artificial intelligence (AI) based on deep learning (DL), to evaluate MRI. Recently, a different spectral/statistical approach has been used to successfully evaluate spatially registered biparametric MRIs for prostate cancer. This study aimed to further assess and improve the spectral/statistical approach through benchmarking and combination with AI. **Methods:** A zonal-aware self-supervised mesh network (Z-SSMNet) was applied to the same 42-patient cohort from previous spectral/statistical studies. Using the probability of clinical significance of prostate cancer (PCsPCa) and a detection map, the affiliated tumor volume, eccentricity was computed for each patient. Linear and logistic regression were applied to the International Society of Urological Pathology (ISUP) grade and PCsPCa, respectively. The R, *p*-value, and area under the curve (AUROC) from the Z-SSMNet output were computed. The Z-SSMNet output was combined with the spectral/statistical output for multiple-variate regression. **Results:** The R (*p*-value)–AUROC [95% confidence interval] from the Z-SSMNet algorithm relating ISUP to PCsPCa is 0.298 (0.06), 0.50 [0.08–1.0]; relating it to the average blob volume, it is 0.51 (0.0005), 0.37 [0.0–0.91]; relating it to total tumor volume, it is 0.36 (0.02), 0.50 [0.0–1.0]. The R (*p*-value)–AUROC computations showed a much poorer correlation for eccentricity derived from the Z-SSMNet detection map. Overall, DL/AI showed poorer performance relative to the spectral/statistical approaches from previous studies. Multi-variable regression fitted AI average blob size and SCR results at a level of R = 0.70 (0.000003), significantly higher than the results for the univariate regression fits for AI and spectral/statistical approaches alone. **Conclusions:** The spectral/statistical approaches performed well relative to Z-SSMNet. Combining Z-SSMNet with spectral/statistical approaches significantly enhanced tumor grade prediction, possibly providing an alternative to current prostate tumor assessment.

## 1. Introduction

Determining the prostate tumor’s potential for growth and metastases is essential for deciding whether a patient should undergo therapy or active surveillance [1,2,3,4,5,6]. Surgery, radiation therapy, and systemic therapy can control the disease but can also be physically, emotionally, and financially debilitating for the patient. The proper treatment choice may prolong life, while preventing unnecessary side effects. Guided [7] and conventional needle biopsy that determines a patient’s status can exact a toll on the patient due to potential hemorrhaging, infection, and pain [8] and possibly incorrectly assessing the patient through needle misplacement [9]. MRI and ultrasound have been used to non-invasively evaluate the prostate, with minimal side effects [10,11,12,13,14]. MRI is particularly sensitive for detecting prostate cancers. MRI scanning generates structural (T2) and functional (diffusion, dynamic contrast enhancement) images that can offer additional insight into the aggressiveness of the tumor [15,16,17,18,19]. Trained radiologists visually inspect the prostate tumor and assess the patient based on specific scoring systems such as PI-RADS [20,21,22,23]. In practice, visual inspection of MRI can be inconsistent, and accurate assessment depends on the experience and training of a given radiologist [21,22,23]. Although injecting contrast reveals the tumor vasculature and provides valuable information, reducing patient inconvenience and simplifying and expediting clinical flow motivate foregoing the contrast scan by scanning with only two parameters diffusion weighted imaging (DWI) that included apparent diffusion coefficient (ADC), and high b-value (HBV), along with the structural imaging T2,. This mode of imaging is called biparametric (BP) MRI [24,25,26,27,28,29,30,31].

Instead of relying on the radiologist’s subjective judgement, quantitative approaches, such as deep learning (DL) and artificial intelligence (AI) algorithms [32,33,34], are applied to BP-MRI to expedite the analysis and aid the radiologist. The DL and AI models were trained on large-scale datasets of the patients to capture spatial features, such as textures, for predicting tumor aggressiveness. There has been great interest in DL and AI throughout technology and science, especially in examining MRI for prostate cancer assessment [32,33,34]. To help sort through the plethora of DL and AI algorithms, a PI-CAI Grand Challenge [35,36,37] evaluated 293 DL and AI algorithms trained on a common BP-MRI dataset of 1500 publicly available cases from 1476 patients with tissue confirmation of the diagnosis. Each of the candidate algorithms produced tumor detection maps and the probability of clinical significance for prostate cancer for each patient. The competing algorithms were evaluated based on an additional, common test set of independent patient data. In particular, a Z-SSMNet algorithm composed by Yuan et al., from Australia [37], achieved a top performing algorithm status in the PI-CAI Grand Challenge for accurate prostate tumor evaluation and detection.

Recently, spectral/statistical techniques have been applied to spatially registered multi- and biparametric MRI [38,39,40,41,42,43] to assess prostate cancer. The techniques were adapted from processing hyperspectral imagers mounted on airborne platforms. Instead of extracting and employing spatial features such as those used in DL and AI, the spectral/statistical method identifies tumors through spectral signatures. A spectral signature is a three-dimensional vector composed of ADC, high b-value, T2 values that distinguish a tumor from normal prostate tissue. This approach is analogous to spectroscopic studies and human color vision, requires minimal training, and can be adapted to different clinical conditions. The pilot studies demonstrated that features such as signal-to-clutter ratio [38,41,42] (a quantitative metric comparing tumor to normal prostate values and discussed in Section 2.6 and Section 2.7), tumor volume [41,43], and tumor eccentricity [41,43] (a quantitative metric describing a tumor’s shape, described in Section 2.12) correlated with tumor grade and showed promise in terms of their ability to assess prostate cancer. Although the area under the curve (AUROC), derived from the receiver operator characteristic (ROC) curve, derived from the spectral/statistical approaches in previous pilot studies was generally not inferior to the results for AI, these studies did not directly compare AI and spectral/statistical approaches at the patient-to-patient level [38,39,40,41,42,43].

DL and AI methods that primarily exploit spatial features such as textures differ substantially from spectral/statistical approaches. These differences may mean that the DL/AI and spectral/statistical approaches are not correlated with each other. Combining spatial and spectral approaches may potentially boost tumor grade prediction, analogous to human vision and its ability to detect and evaluate a given scene.

## 2. Materials and Methods

This is a retrospective study comparing two approaches for assessing prostate cancer from biparametric MRI. The novel techniques employed are summarized below, with greater detail in prior reports and small-cohort pilot studies [37,38,39,40,41,42,43]. Some details are also summarized in subsequent sections of this paper.

### 2.1. Overall Approach

**Figure 1:**
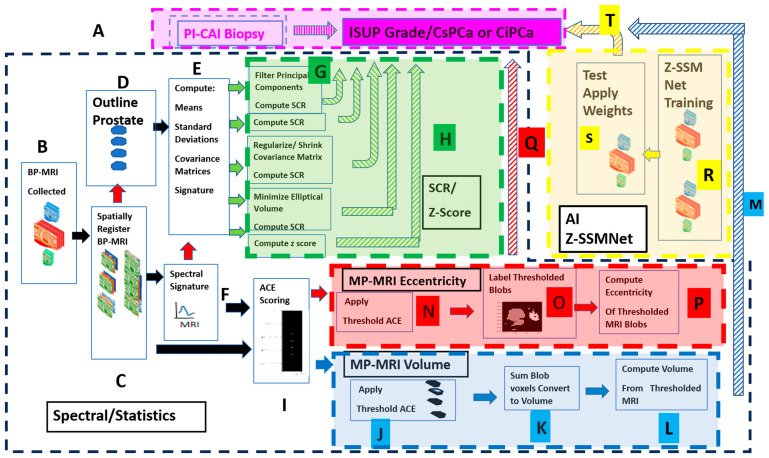
Schematic showing the overall process for calculating features from spectral/statistical approaches and the Z-SSMNet algorithm and their relationship to ISUP and the detection map. The PI-CAI result provides the “ground truth”, or prostate cancer evaluation (Label A). The spectral/statistical approach (Labels B through Q) assembles (Label B) the spatially registered BP-MRI (Label C), from which spectral signatures (Label F) and prostate organ outline (Label D) are generated, and provide input (Label E) for the SCR (Label G), tumor volume (Labels J through M), and tumor eccentricity (Labels N through Q). These features are compared to the PI-CAI ISUP and CsPCa. The Z-SSMNet (Labels S, R, T) predictions and tumor outlines for patients in fold 0 are generated from weights (Label S) taken from training (Label R) with the folds 1, 2, 3, 4.

Figure 1 shows the overall scheme to compare a metric related to the Gleason score, namely, the International Society of Urological Pathology (ISUP) grade [44], and clinically significant (insignificant) prostate cancer, or CsPCa (CiPCa), with metrics generated from spatially registered MRI, namely the z-score and processed signal-to-clutter ratio (SCR) [38,41,42] (green-highlighted panel discussed in Section 2.6 and Section 2.7), eccentricity (red-highlighted panel, a quantitative metric describing tumor shape and discussed in Section 2.12), and tumor volume (blue-highlighted panel, discussed in Section 2.10). In addition, an AI approach was applied to the same data (yellow panel). ISUP grade is determined from PI-CAI pathology analysis [44] of the histopathology slides from MRI-directed biopsy (MRBx), systematic biopsy (SysBx), the combination of MRBx and SysBx, and radical prostatectomy (RP) (Figure 1, label A) [45]. For this study, patient MRI data and their assessments were gathered as part of the PI-CAI Grand Challenge [35,36,37] (Figure 1, label A, B). In the BP-MRI arm [38,39,40,41,42,43] of the study, a spatially registered vectorial 3D image to the voxel level is assembled from the individual MRI sequences, specifically the apparent diffusion coefficient (ADC), high b-value (HBV) from the diffusion weighted images (DWI), and T2 by translation, resizing the images (Figure 1, label C). Using the spatially registered MRI, the prostate is manually outlined to generate a normal prostate mask (Figure 1, label D), input for covariance matrix computation (Figure 1, label E). In-scene signatures (Figure 1, label F) are derived from the spatially registered vectorial 3D image to provide input for the z-score and SCR computation [38,41]. Noise in the SCR [38,41] is reduced through principal component filtering, regularizing the covariance matrix (Figure 1, label G). The processed SCR and z-score are linearly (logistical probability) fitted to the ISUP grade (CsPCa/CiPCa), respectively (Figure 1, label H, green hashed arrows). Metrics describing the linear and logistic fits are given by the correlation coefficients (R) and the AUROC from the receiver operator characteristic (ROC) [38,39,40,41,42,43].

Figure 1 also summarizes the procedures for generating the eccentricity and tumor volume measurements. Adaptive cosine estimator (ACE) detection (a spectral conical decision surface based on a spectral signature that distinguishes tumor and normal tissue, discussed in Section 2.9) calculations (Figure 1, label I); thresholding (Figure 1, label J and N) and prostate volume calculations (Figure 1, label K and L); prostate tumor eccentricity (Figure 1, label O and P); and linear regression and logistic probability are fit (Figure 1, label M) to the ISUP and CsPCa results obtained from the biopsy (Figure 1 label P). The direction of the output data to be used as input is denoted by arrows. Red arrows and boxes indicate eccentricity calculations from BP-MRI-based data; blue arrows and boxes denote tumor volume estimated from biparametric MRI.

The spatial registration of the biparametric data, processing, and calculations of the SCR, ROC curve, and AUROC were executed using the Python 3 programming language, scikit-learn, numpy, panda libraries.

In addition, a deep learning approach, entitled Z-SSMNet (zonal-aware self-supervised mesh network), developed by Yuan et al. [37], was applied to the same 42-patient cohort. The 42 patients resided in the validation set of fold0, designated by the PI-CAI [36]. The Z-SSMNet algorithm was trained on the rest of the data, composed of the validation set of fold1, fold2, fold3, and fold4, to generate weights (Figure 1, label R) in order to infer likelihood and detection maps for the 42 patients (Figure 1, label S). These inferred values were used in the regression fits (Figure 1, label T).

**Figure 2:**
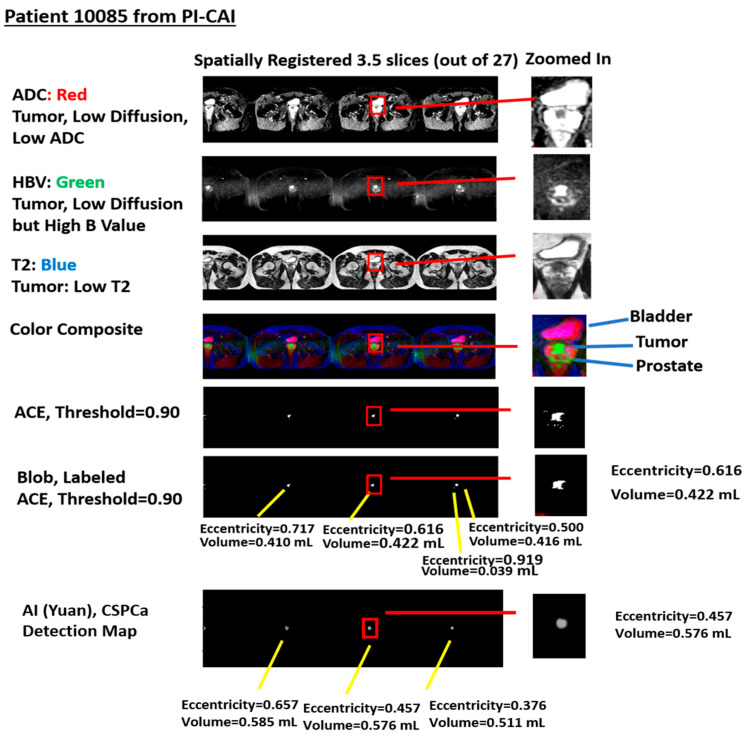
Example of stitched (four slices stitched together) spatially registered biparametric MRI, the Z-SSMNet detection map, the color composite, and the blobbing, labeled according to the spectral/statistical approach. Blob eccentricity and volume computations for the spectral/statistical and Z-SSMNet detection maps. ADC is assigned red, HBV is assigned green, and T2 is assigned blue. In the color composite, the tumor appears as green. ACE output (ranges from 0.0 to 1.0) with threshold 0.90.

Figure 2 shows a specific example taken from patient 10085 in the PI-CAI data collect. The figures show four spatially registered slices that have been stitched together. Selected stitched sections of ADC, high b-value (HBV), and T2 files were assigned red, green, and blue colors to form the color composite. In the color composite, the tumor appears as green. Also shown, an image resulting from applying a threshold (set to 0.9) to stitched selections from the adaptive cosine estimator (ranges from 0.0 to 1.0) along with the labeled blobs and their volume and eccentricity. In addition, Figure 2 shows the computed volumes and eccentricity for the blobs from the Z-SSMNet (Yuan) detection map.

### 2.2. Study Design and Population

MRI and assessments were collected and stored using PI-CAI [35,36,37]. The PI-CAI challenge stores an annotated multi-center, multi-vendor publicly available dataset of 1500 BP-MRI exams that includes clinical and acquisition variables. Various histopathology techniques were used [35,36], but only a subset of the 1500-patient study underwent biopsy or had available biopsy results. Patients were scanned at a variety of centers using assorted Siemens and Philips scanners. The PI-CAI data collection [35,36] only includes biparametric MRI, namely ADC, HBV, and T2 sequences.

Previously, 42 consecutive patients who had been biopsied in the PI-CAI database were assessed. All patients exhibited biopsy-proven adenocarcinoma of the prostate, with a mean patient age of 65.1 years (range, 50 to 78 years), a mean PSA of 13.49 ng/mL (range, 1.5 to 81.95 ng/mL), a mean prostate volume of 60.6 cm^3^ (range 19 to 192 cm^3^), and a mean ISUP grade of 1.12 (range, 0 to 5); 31(11) cases were confirmed as clinically insignificant (significant) prostate cancer. This study placed no restrictions on tumor location within the prostate. Informed consent was exempted due to the retrospective nature of this study. All cases were anonymized for subsequent analysis.

### 2.3. Spatial Registered Vectorial 3D Image Assembly: Magnetic Resonance Imaging

The biparametric MRI data collected [35,36] were composed of structural (T2) images, i.e., DWIs, specifically, the ADC and HBV. This data collection excluded DCE images.

### 2.4. Spatial Registered Vectorial 3D Image Assembly: Image Processing and Pre-Analysis

Before applying spatial registration, the spatial resolution and spatial offsets for the scanning setup of a given patient were read from image header files for each of the MRI sequences (ADC, HBV, and T2). The MRI images were digitally resized [38,39,40,41,42,43] to the sequence with the lowest spatial resolution in the transverse direction. Using the offsets listed in the image header files, a few pixels (or no pixels) of the images were translated to the reference image (ADC and HBV). Based on the known location of the axial offsets, the slices were selected and resampled to match the offsets. Also, small transverse translation adjustments, based on visual inspection, were applied to the T2 image to match the appropriate ADC and HBV slices.

A “cube” is composed of stacked individual slices that had been appropriately scaled, translated, cropped, and spatially registered at the voxel level. Following cropping, all images shared the common field of view (FOV). These “three dimensional” (two transverse directions, plus a spectral dimension composed of ADC, HBV, and T2 images) cubes were “stitched” together into a narrow three-dimensional vectorial image cube to depict the entire body within the common field of view of the MRI scan. This stitching of MRI slices (or mosaicking) emulates configurations used in remote sensing, in which small patches are stitched together into large swaths of areas, thereby increasing the processing speed to handle large, high-dimensional data. The spatial registration for each patient took a few seconds to process on a Windows 10, base speed 2 Ghz, cache memory 8 Gbyte machine. The registration was visually inspected. Sometimes, small translation (1 voxel) corrections were applied for individual slices to correctly spatially register the BP-MRI, along with cropping to ensure a common size for all slices within the stack.

### 2.5. Overall Quantitative Metrics Description: SCR and Z-Score

In medical practice, trained radiologists visually inspect multiple MRI images to qualitatively determine tumor aggressiveness [22,23]. In contrast, the SCR and z-score are quantitative metrics denoting a tumors’ departure from normal prostate tissue. The z-score and SCR formulation combine information from all BP-MRI sequences. The SCR and z-score compute the difference between the mean tumor signature value and the mean normal prostate value, scaled by the normal prostate standard deviation for each MRI sequence (ADC, HBV, and T2). However, the z-score does not account for correlation among the BP-MRI sequences (ADC, HBV, and T2). Instead, the SCR decorrelates the sequences by whitening the spatially registered BP-MRI. In the whitening process, noise is added. The SCR computation requires computing the covariance matrix that ultimately corrects for correlations among the different sequences (i.e., the correlation between ADC and DWI) and thereby determines the true contribution of each sequence. References [38,39,40,41,42,43] summarize the mathematics behind the SCR algorithm. For each patient, SCR calculations took a few seconds to process on a Windows 10, base speed 2 Ghz, cache memory 8 Gbyte machine.

### 2.6. SCR: Filtering Noise

The covariance matrix for the SCR can be decomposed into principal components (PC) [46]. Principal components are linear combinations of all MRI components. The principal components are orthogonal to each other and therefore, decorrelated. Conventionally, the ordering of the principal components is based on their eigenvalue or statistical variation. The high eigenvalue (low PC number) PC image displays high variation within the image. In contrast, noisy images (high PC number) are associated with principal components having small eigenvalues and low variation within the image. Filtering and eliminating the noisy (low eigenvalue, high PC number) principal components reduces noise from degrading the covariance matrix inversion and increases the SCR calculation accuracy. References [38,39,40,41,42,43,47] summarize the mathematics for filtering principal components.

### 2.7. Regularization and Shrinkage

Regularization [39,42,48] results from the constraints on the coefficients in the Langrangian optimization process. In this application, regularization also ensures that the covariance matrix follows a normal distribution. The analytic formula only approximates the covariance matrix. Shrinkage regularization [39,42,48] perturbs the original covariance matrix CM(γ) by mixing a diagonal matrix with an adjustable parameter γ to generate a regularized or modified regularized covariance matrix. The appropriate γ minimizes the discriminant function and thereby, more appropriately mimics the normal distribution. Regularized and modified regularized covariance matrix calculations follow the same procedure but differ in their choice of the mixing diagonal matrix. References [39,42,48] summarize the mathematics behind regularization procedures.

### 2.8. Logistic Regression

A logistic regression fit [49] results from fitting the processed SCR, z-score, patient data, and Z-SSMNet likelihood scores to the dependent categorical variable CsPCa. The ISUP grade is taken from the biopsy. The clinically significant PCa (CsPCa) was assigned to ISUP grade ≥ 2. The clinically insignificant PCa (CiPCa) was assigned to <2. New randomized sets were generated 1000 times, forming configurations of patients within the training/test sets, generating 1000 ROC [50] curves and resulting in a distribution of AUROC. The distribution of AUROC scores was recorded, along with the 2.5% and 97.5% largest AUROC, delineating the 95% confidence interval. The fit quality was assessed through the AUROC and the 95% confidence interval from the ROC curves.

### 2.9. Adaptive Cosine Estimator (ACE) Algorithm

The adaptive cosine estimator (ACE) algorithm is a supervised target detection algorithm [38,39,40,41,42,43,51]. Supervised target detection algorithms [38,39,40,41,42,43,51] peruse and classify a voxel into either a target (prostate tumor) or background (normal prostate), based on information about the target (tumor), specifically the tumor signature. The tumor signature S is a three-dimensional vector whose components are intensity values within the manifold (ADC, high b-value, T2) that characterize the target. The background is characterized by a mean three-dimensional vector m and a covariance matrix CM (three dimensions X three dimensions) that includes the variance and accounts for correlations among the different dimensions. A multi-dimensional (3-D for BP-MRI) cone is centered around the target signature S. The ACE decision surface follows this cone. Voxels whose ACE scores reside within the decision cone are assigned to the target. Voxels residing outside the cone are assigned to the background. References [38,39,40,41,42,43,51] offer a more detailed summary of the ACE algorithm and clarifying equations.

### 2.10. Tumor Volume Measurements and Supervised Target Detection

To compute a metric associated with the tumor volume, the ACE algorithm was applied to the spatially registered BP-MRI [40,41,43]. Voxels that lay inside the decision cone or exceed a threshold for ACE scores were assigned to the tumor. Normal tissue was assigned to voxels that resided outside the decision cone or had ACE scores residing below the threshold. The number of voxels exceeding a given threshold were counted and assigned tumor status. This sum is converted to volume based on the MRI spatial resolution. References [40,41,43] summarize the mathematics behind the tumor volume computation.

### 2.11. Labeling and Blob Generation

Within computer vision, connected component labeling, or blobbing and labeling [40,41,43], refer to the process of objectively aggregating neighboring voxels. The blobbing is applied to a mask image or binary image following the application of a threshold to the ACE detection image. The values of 1 or 0, or “true” or “false”, are associated with the tumor (background) in each masked image. Each “true” voxel peruses voxels within a given neighborhood (1 voxel away) to see if they are also “true”. Blobbing is associated with an 8-pixel connected neighborhood involving the “true”, “1,” or “tumor” voxels in the masked image. If the “true” voxels are connected, the particular voxels are collected and labeled as a member of a blob. Blobs smaller than 5 voxels (~10^−2^ mL) were filtered out.

### 2.12. Eccentricity Calculation

Custom software coded in Python 3 calculated the eccentricity [40,41,43] for every labeled blob. Following the identification of a blob, the moment of inertia matrix I for the kth blob was computed. The eigen equation (using the moment of inertia I) was solved, resulting in computing the eigenvalues for each blob. The largest eigenvalue was assigned to the large axis l_k_, and the second eigenvalue was assigned to the transverse moment s_k_. The eccentricity E_k_ for the kth blob is a normalized difference between the major axis l_k_ and minor axis s_k_. Eccentricity values E_k_ range from 0 (spherical shape) to 1 (line). References [40,41,43] offer a more detailed summary of the eccentricity computation and clarifying equations.

### 2.13. Machine Learning Application: Z-SSMNet

This study directly compares the findings of the spectral/statistical techniques with the application of a publicly available trained AI model derived from a highly performing program, as judged in the recent PI-CAI Challenge [35,36,37]. Specifically, this study examined and applied the zonal-aware self-supervised mesh network (Z-SSMNet) algorithm, developed by Yuan et al. from Australia [37].

The Z-SSMNet algorithm [37] used axial-plane image sequences from the BP-MRI PI-CAI dataset. No clinical variables from routine practice (patient age, PSA, prostate volume) nor acquisition variables (MRI scanner manufacturer, vendor, scanner, model, b-value of the diffusion weighted imaging, etc.) associated with the imaging were employed to guide the prediction. The Z-SSMNet algorithm ameliorated some issues that plague DL, resulting in improved performance. Current state-of-the-art AI algorithms are often based on deep learning for 2D images that fail to capture inter-slice correlations in 3D volumetric images. The 3D convolutional neural networks (CNNs) partly overcome this deficiency. However, 3D CNN does not handle the anisotropy within images and can introduce artifacts. In addition, due to the limited amount of labeled data for BP-MRI, along with labeling difficulties, CNNs employ relatively small datasets, leading to poor performance. To address these limitations, a new Z-SSMNet model was deployed on the 3D nnU-Net framework [52,53] to help learn region-specific high-level semantic information, gain zonal specific domain knowledge, (whole gland or the transitional and peripheral zones), to constrain the computation and improve the diagnostic precision for csPCa, and to reduce false positive detection. The new method adaptatively fuses multiple 2D/2.5D/3D CNNs following the U-Net architecture [54] and adjusts for both sparse inter-slice information and dense intra-slice information in BP-MRI. In addition, a self-supervised learning (SSL) technique extracts textures and boundary information and pre-trains the unlabeled data in order to extract the generalizable image features. Experiments on the PI-CAI Challenge BP-MRI datasets show high performance for csPCa detection and diagnosis. The AUROC and average precision (AP) scores are 0.890 and 0.709 in the hidden validation and tuning cohort (100 cases) (2nd rank) and 0.881 and 0.633 in the hidden testing cohort (1000 cases) (1st rank), respectively.

**Figure 3:**
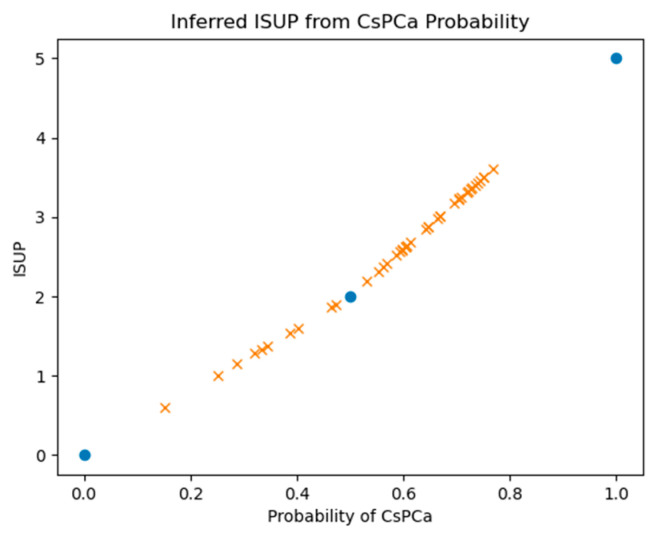
Calibration plot for interpolating ISUP values from Z-SSMNet probability of CsPCa, based on linear calibration curves. Blue circles denote known fixed points.

The Z-SSMNet algorithm [37] computes a suspicion score (single floating-point value between 0–1) for a given patient, representing the likelihood that a patient harbors a clinically significant cancer. The relationship between CsPCa likelihood and ISUP is unclear. The ISUP grades 0, 2, and 5 should correspond to probabilities of 0., 0.5, and 1.0, respectively. In addition to recording Z-SSMNet’s likelihood scores for each patient, this study inferred the ISUP by interpolating the linear relationship. Figure 3 plots the inferred ISUP grades against the probability of CsPCa and shows the known fixed points (blue filled circles) and the patient values derived from linear interpolation. This study also interpolated patient values based on ISUP = 3.6409*tanh^−1^ (probability), where 3.6409 is chosen so that probability = 0.5 corresponds to ISUP = 2.

The 42 cases analyzed using the spectral/statistical approach were extracted from the validation set of fold0 (semi-supervised setting) in the PI-CAI Grand Challenge. The weights for the inference were based on the model trained on the rest of the data, composed of the validation set of fold1, fold2, fold3, and fold4.

### 2.14. Univariate and Multivariate Fitting

One or multiple independent variables are fitted to a single independent variable using univariate and multivariate linear regression analysis, respectively [54,55]. In this study, the independent variables correspond to an individual (such as the largest) and collective (such as the average or weighted) blob eccentricity, blob volume derived from fixed data, thresholds, signal-to-clutter ratio (PC filtered, regularized) ACE, and likelihood scores from Z-SSMNet. The dependent variable is the ISUP grade or CsPCa. The latter is a categorical variable (binary variable, or either true or false). The fits minimize the error in a least-squares calculation by finding the optimal fitting coefficient for the independent variable. These fitting coefficients can be applied to the independent variables to generate a fit, which are then compared to actual data. Correlation coefficients and fitted lines test the agreement of the computed *p* values to assess the probability that the fit is or is not correlated. Confidence intervals are computed for every variable, along with *p* values for the multivariable fit. Reference [27] offers a more detailed summary of the linear regression fitting and provides clarifying equations.

Tumor aggressiveness or CsPCa estimation might be improved (beyond univariate regression) by applying linear multi-variate [55,56] or logistic probability regression (Section 2.8) [49] to the combination of the AI approach with spectral/statistical algorithms. Previously [21,22], SCR combined with tumor eccentricity, volume, increased the correlation coefficient, AUROC.

## 3. Results

**Figure 4:**
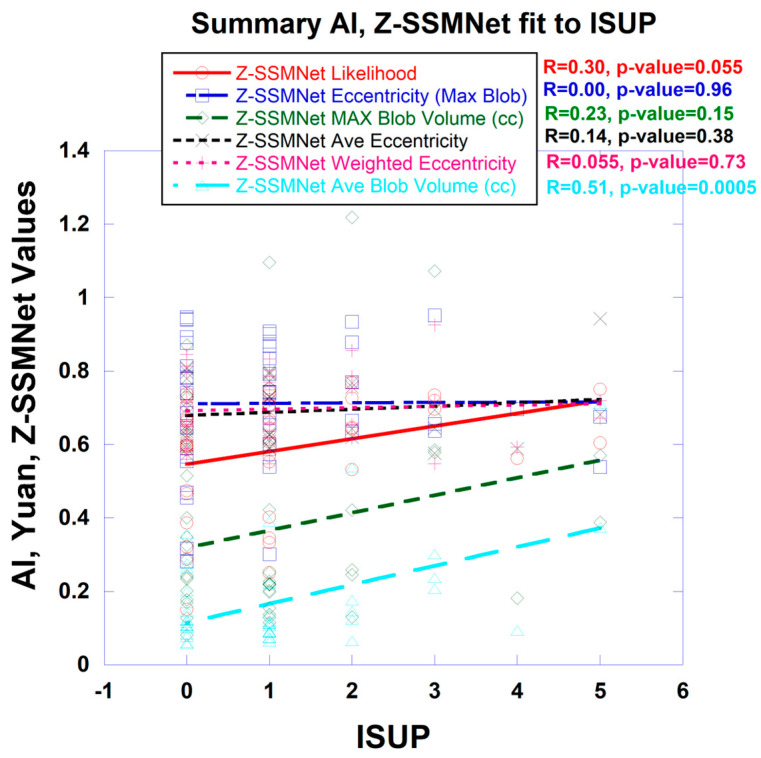
Plot of features of individual patients computed from Z-SSMNet detection map plottted against ISUP values. Correlation coefficients (R) and p-values from linear regression shown.

Yuan’s algorithm generates likelihood scores and detection maps. Figure 4 plots a variety of measures (such as the likelihood) derived from the Z-SSMNet algorithm computed from each patient and from the computed detection map. Processing of the detection map generated a number of features, specifically, the number of blobs, eccentricity from the largest blob, average eccentricity, mass-weighted eccentricity, maximum blob volume, average blob volume, and total tumor volume. The likelihood and the detection maps features were plotted against the ISUP score derived from biopsy. From linear regression, the correlation coefficient (R) and *p*-value are also shown in the figure. Unlike spectra/statistical approaches, the AI-derived tumor eccentricity was not a good predictor of prostate tumor aggressiveness.

The Z-SSMNet likelihood and features derived from the detection map were plotted and fitted through linear regression to the ISUP score for each patient in the 42-patient cohort. The R (*p*-value) and AUROC [95% confidence interval] from the Z-SSMNet (Yuan) algorithm relating the ISUP to PCsPCa is 0.298 (0.06) and 0.50 [0.08–1.0], relating it to average blob volume is 0.51 (0.0005) and 0.37 [0.0–0.91], relating it to total tumor volume is 0.36 (0.02) and 0.50 [0.0–1.0], relating it to eccentricity (maximum blob) is 0.0 (0.96), relating it to eccentricity (average) is 0.14 (0.38), and relating it to eccentricity (weighted) is 0.06 (0.73). From earlier studies, the R (*p*-value) from the spectral/statistical approach relating the ISUP score to the processed SCR ranged from 0.55 to 0.58 (<<0.02), relating it to tumor volumes ranged from 0.37 to 0.42 (0.018 to 0.03) and 0.70 to 0.95 [0.33–1.0], and relating it to tumor eccentricity (largest blob) ranged from 0.35 to 0.37 (0.01 to 0.015) and 0.44 to 0.90 [0.12–1.0]. Generally, the R and AUROC values from the Z-SSMNet algorithm are lower than those from the spectral/statistical approaches.

Table 1 summarizes the best performing univariate linear and logistic regression from Z-SSMNet predictors to ISUP and CsPCa, respectively.

AI-derived features and spectral/statistical features were combined in a multi-variable fitting to ISUP. The best performing multivariate linear regression fits are summarized in Table 2. The biggest increase in correlation occurs for features that mix AI and spectral/statistical approaches, which also yields the lowest cross-correlation coefficient from mixing AI and spectral/statistical approaches. It should be noted that combining the z-score and SCR (modified regularization) from the spectral/statistical approach shows high univariate R1 and R2, as well as high cross correlation, but poor R12 relative to the combinations that mix AI and spectral/statistical approaches. AI average blob size and SCR results in R-0.70, *p* < 0.02, significantly higher than multiple regression fits involving AI or the spectral/statistical approach alone. Combining the spectral/statistical approach-derived eccentricity (maximum blob), average blob volume, or maximum blob with the Z-SSMNet-derived average blob volume in a multi-variate fit to ISUP resulted in minimal increases in the correlation coefficient (R12) due to relatively poor univariate correlation (R1, R2) and high cross-correlation among the tumor volume measures. Previously, univariate logistic fits of z-scores and processed SCR from the spectral/statistical method achieved AUROC scores of 1.0 [1.0–1.0] [43], resulting in similar AUROC scores for multi-variate logistic fits that include SCR derived from the spectral/statistical approach.

**Figure 5:**
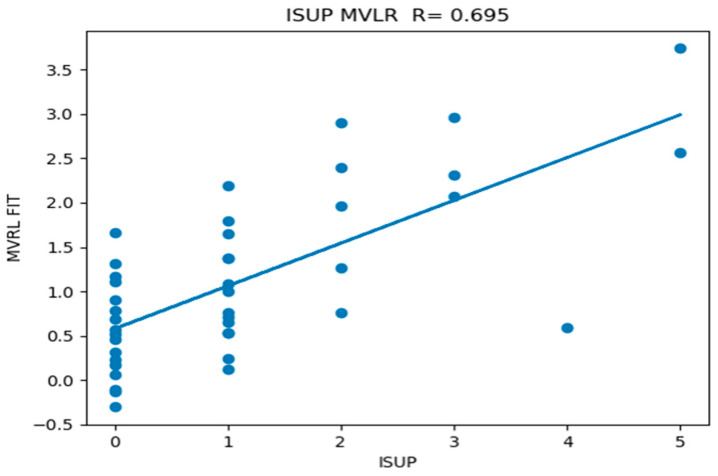
Plot of multi-variate fit to ISUP combining average AI tumor blob volume and spectral/statistical SCR from modified regularization.

Figure 5 plots the individual patient data, as well as the calculated multi-variate fits taken from the average blob derived from AI (Z-SSMNet) and the modified regularized SCR from the spectral/statistical approach against the ISUP derived from biopsy.

## 4. Discussion

This is the first study to directly compare and combine DL/AI algorithms and spectral/statistical approaches on a per patient basis. This retrospective pilot study of 42 patients from the PI-CAI data collection shows that the new spectral/statistical approach demonstrates performance at least comparable to that of a DL/AI algorithm for achieving high correlation with tumor grade and accurately predicting clinically significant prostate cancer. The high performance of spectral/statistical approaches is notable due to its simplicity in conception, calculation, application, and understanding relative to AI. Unlike spectral/statistical approaches, the eccentricity from the DL/AI algorithm detection map did not show anti-correlation with prostate tumor grade. Based on the results of this pilot study, further studies to confirm these promising results regarding the relative efficacy of individual spectral/statistical and AI approaches, along with the combination of the two methods, are warranted.

Studies of adenocarcinoma morphologies of breast cancer [56] and lung cancer [57,58] show that the tumors become more symmetrical as the tumor grade increases, or that adenocarcinoma eccentricity is anti-correlated with tumor grade. Recently [40,41,42,43], evidence has been gathered showing that prostate cancer, nearly always an adenocarcinoma, is also anti-correlated with tumor grade. One recent study [40] examined outlined histopathology slides from prostatectomy, and spectral/statistical approaches were applied to 26 patients with multi-parametric MRI [40] and 42 patients with biparametric MRI [41]. In this current study, DL/AI found that tumor grade correlated with tumor volume but did not correlate with tumor eccentricity or shape. The detection maps generated by the models in the PI-CAI challenges may not accurately predict the tumor region, as the evaluation criterion is that the overlap between the predicted tumor and the true value exceeds 0.1. The DL/AI algorithms may further benefit from the incorporation of training using tumor morphology, such as tumor volume and eccentricity, instead of confining their training to spatial textures for predicting the clinical significance of prostate cancer.

This study only analyzed 42 consecutive patients. All the studied patients resided in the PI-CAI fold 0 among five folds of the cross-validation study in the PI-CAI Grand Challenge. Although the calculated *p*-values for correlating the ISUP and the 95% confidence intervals for the AUROC in the ROC curves achieved highly statistically significant values, additional independent study using a higher number of patients are needed to meaningfully confirm the results.

This study confined its analysis to only one DL/AI algorithm, namely the Z-SSMNet algorithm by Yuan et al. from Australia [37]. The Z-SSMNet algorithm achieved second place (among 293 algorithms) in the PI-CAI Grand Challenge assessment. There is no evidence that the choice of the Z-SSMNet algorithm achieves a performance inconsistent with other high-achieving performers in the Grand Challenge. Nevertheless, future studies should process the selected patient cohort using additional AI algorithms.

For multi-variate regression fitting, combining DL/AI with spectral/statistical approaches achieves a higher correlation with tumor grade than does separately applying regression involving DL/AI or spectral/statistical approaches. The low correlation coefficient should be noted, meaning that there is less replication and possibly, more synergy, among algorithms that are distinctly different. Further investigations into the combined use of DL/AI and spectral/statistical approaches are warranted, including a larger number of patients and employing other DL/AI algorithms.

The DL/AI and spectral/statistical approaches may possibly be deployed in areas beyond grading the primary prostate tumor. Quantitative analysis of MRI may be used in the active surveillance of prostate cancer patients through multiple MRI scanning, evaluating, and grading measures over time to see if the disease is progressing beyond clinical insignificance and therefore, requiring therapy [59]. Similarly, MRI can monitor the prostate cancer patient following surgery, and possibly during chemotherapy and radiation therapy, to check for response to treatment and disease recurrence [60]. These same techniques can be used to detect metastatic spread to nearby lymph nodes [61] and to more distant organs through whole-body MRI scans and integration with positron emission tomography [11]. Additional studies can and should examine the role of DL/AI and spectral/statistical approaches in complementing and supplementing clinical analysis in resolving clinical difficulties and complications [62].

Human vision employs both spatial and spectral features to detect and discriminate among targets. This study showed that combining spatial and spectral approaches can be especially effective in determining prostate tumor aggressiveness. Emulating vision (or possibly human-ideation-driven approaches), instead of intelligence, may prove a more fruitful avenue for improving algorithmic performance for analyzing prostate tumors depicted in MRI.

## 5. Conclusions

This first pilot retrospective study compared and combined prostate tumor assessments from DL/AI algorithms with spectral/statistical approaches. The spectral/statistical approaches performed well relative to DL. Combining AI with spectral/statistical approaches significantly enhanced tumor grade prediction.

## Figures and Tables

**Table 1:** AI (Z-SSMNet) univariate linear and logistic regression summary.

	R	*p*-Value	AUROC [2.5–97.5% CI]
Independent Variable			
Probability of CsPCa	0.298	0.0554	0.503 [0.083–1.0]
ISUP (Linear Conversion)	0.301	0.0525	0.503 [0.083–1.0]
Average Blob Volume	0.512	0.00053	0.367 [0.0–0.909]
Total Volume	0.355	0.021	0.501 [0.0–1.0]

**Table 2:** Summary of uni- and multi-variate fit to ISUP, combining AI and the spectral/statistical approach.

Independent Variable 1 (AI Mostly)	R1 (Uni-Variate)	Independent Variable 2 (Spectral/Statistical Only)	R2 (Uni-Variate)	Variable 1-Variable 2 Cross-Correlation	R12 (Multi-Variate)	Probability (F-Statistic)
z-score	0.532	SCR (Modified Reg)	0.57	0.985	0.595	0.0002
Ave Blob Volume (AI)	0.512	SCR (2 PC Removed)	0.554	0.412	0.635	0.000042
Ave Blob Volume (AI)	0.512	z-score	0.532	0.167	0.683	0.0000059
Ave Blob Volume (AI)	0.512	SCR (Reg)	0.588	0.384	0.665	0.000012
Ave Blob Volume (AI)	0.512	SCR (Modified Reg)	0.57	0.219	0.695	0.0000026

## Data Availability

The data presented in this study are available from the PI-CAI Grand-Challenge at https://pi-cai.grand-challenge.org/DATA/ (accessed on 4 February 2025). Additional raw data supporting the conclusions of this article will be made available by the authors on request.

## Abbreviations

The following abbreviations are used in this manuscript:

SSA	spectral/statistical approach
SCR	signal-to-clutter ratio
ACE	adaptive cosine estimator
ADC	apparent diffusion coefficient
AUROC	area under the curve
AP	average precision
BP-MRI	biparametric MRI
CsPCa	clinically significant prostate cancer
DWI	diffusion weighted imaging
HBV	high B-value
ROC	receiver operator characteristic
Mod Reg	modified regularization
Reg	regularization
PC	principal component
DL	deep learning
AI	artificial intelligence
ISUP	International Society Urological Pathology
PI-RADS	Prostate Imaging Reporting and Data System
PI-CAI	prostate imaging artificial intelligence
Z-SSMNet	zonal-aware self-supervised mesh network
